# A 3D Visualization Method for Bladder Filling Examination Based on EIT

**DOI:** 10.1155/2012/528096

**Published:** 2012-12-31

**Authors:** Wei He, Peng Ran, Zheng Xu, Bing Li, Song-nong Li

**Affiliations:** State Key Laboratory of Power Transmission Equipment & System Security and New Technology, Chongqing University, Chongqing 400030, China

## Abstract

As the researches of electric impedance tomography (EIT) applications in medical examinations deepen, we attempt to produce the visualization of 3D images of human bladder. In this paper, a planar electrode array system will be introduced as the measuring platform and a series of feasible methods are proposed to evaluate the simulated volume of bladder to avoid overfilling. The combined regularization algorithm enhances the spatial resolution and presents distinguishable sketch of disturbances from the background, which provides us with reliable data from inverse problem to carry on to the three-dimensional reconstruction. By detecting the edge elements and tracking down the lost information, we extract quantitative morphological features of the object from the noises and background. Preliminary measurements were conducted and the results showed that the proposed algorithm overcomes the defects of holes, protrusions, and debris in reconstruction. In addition, the targets' location in space and roughly volume could be calculated according to the grid of finite element of the model, and this feature was never achievable for the previous 2D imaging.

## 1. Introduction

Bladder filling causes the desire to urinate when the bladder contains a certain volume of urine. But for unconsciousness elders, some handicapped with spinal cord injury or patients with urological disease, this sense will not occur. Urinary incontinence or lack of bladder control is an embarrassing problem, in case that many patients need professional nursing. And the work of nursing may be greatly reduced if the urination is detected and alarmed in time. In clinical, the traditional method to solve this problem is draining urine out by a catheter inserted in the bladder. But the intubation is invasive and not suitable for most patients, because it may cause secondary infection of the urinary tract. A way for measure in real time is the ultrasound imaging. Researchers have developed ultrasound bladder volume measurement devices to evaluate bladder volume. However, these devices are inconvenient for continuous monitoring, moreover, the ultrasonic images are greatly influenced by the human intraperitoneal gas [1, 2].

Several investigators over the last 20 years have verified that the electrical properties of human tissues and body fluids are significantly different and have demonstrated that measurement of these properties has obvious clinical potential [3]. Electric impedance tomography distills biomedicine information without trauma and generates real-time image, which examination is not necessary straight line to avoid the intra-peritoneal gas affection. Consequently, this technology is applied to measure and visualize impedance changes in bladder. 

As we know, the filling bladder is located just a little beneath the lower abdomen. On account of different physiological and structural characteristics of patient's abdomen, traditional closed EIT system is difficult to meet in different shape and location of focus detection [4]. Therefore, we chose planar array EIT system due to its convenience in operation [5]. In order to solve the problem of lack in effective precondition in the past researches of 3D volume estimation, we propose a system of 64 electrodes rectangular array with adaptable combination mode of injection and measurement [6]. This system has features of adjustable multifrequency, high accuracy, and being portable and flexible in application, which is quite suitable for long-term clinical monitoring via appropriate upgrade.

Another reason that restricts the 3D EIT development is the lacking of proper algorithms. The previous algorithms applied in EIT included Filtered Back-projection [7], Spectral Expansion method [8], Newton's one-step error reconstruction [9], Genetic Algorithms [10], and Weighted Minimum Norm method [11], most of which are confronted with the severely ill-posed problem and the great amount of calculation. Through the analysis of the respective advantage and disadvantage of Tikhonov [12] and NOSER regularizations [9], we developed the combined regularization algorithm of expectable spatial resolution for 3D EIT, which ensures more uniform impedance estimation and deeper investigation depth.

Since the boundary of the targets or structures are usually contained in the cell of the three-dimensional image, the detection and reconstruction of the edge surfaces from the reconstructed electrical impedance are mong of the important research issues in three-dimensional image analysis [13]. The isosurface is a common approximation of the boundary surface within biomedical images. However, a fixed values iosurface is not suitable in approximating the boundary surface in EIT inverse problems due to large errors of the results [14]. To adapt the local difference of the complex boundary surface, we have improved the work of the literatures [15]. The formation of new method, by which, constructs the adaptively approximating to the boundary surface of the targets with different surface patches in different local regions. Consequently, the approximation accuracy has been considerably improved.

## 2. Materials and Methods

### 2.1. System Description

**Figure 1:**
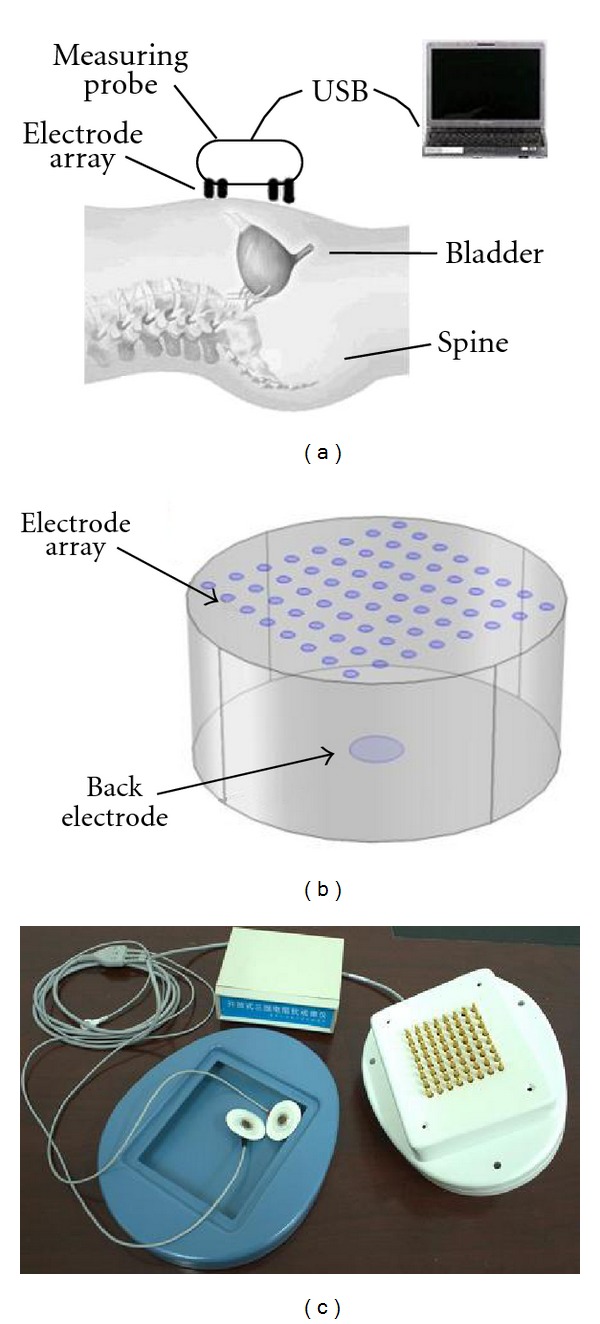
(a) System measurement, (b) Electrodes arrangement, and (c) Experimental Prototype.

On account of different physiological and structural characteristics of patient's waist, the open EIT system in Figure 1(a), by simply placing the measuring probe onto the targets, could make the measurement and avoid the trouble of routine electrode pasting. The measuring probe is an 8 × 8 electrode array with a back electrode as the signal ground is placed on the patient's back to make the current evenly distributed into the body for deeper detection. The measurement and reconstruction field are the area between the electrode array and the back electrode.

As in examination, a sinusoidal current is injected from the 64 electrodes in turn and outflows from the back electrode in Figure 1(b). The measurements are taken from the rest 63 electrodes. In each examination, we will obtain 64 × 63 = 4032 independent measurements from excitation, which greatly increased the amount of available data comparing with most current reported methods, as the 32 electrodes are in round for chest examination (maxim 32∗31 = 992 measurements) [16] and the fixed voltage source with 16∗16 measuring points for breast cancer detection [17].

This system has features of constant current source, good antijamming capability, low output impedance, and deep detection area, which is supplied by medical power and communicates with notebook via USB as in Figure 1(c). While used for long-term monitoring, the device can be replaced with lithium battery powered, Bluetooth communication and belt-contact electrode array, which enables the application for inpatients or even patients at home.

### 2.2. EIT Inverse Problems

The inverse problem is the process of calculating the internal conductivity distribution based on the boundary voltage. EIT image reconstruction is a nonlinear ill-posed problem, and it is only to deduce the impedances in the measurement filed by approximation. In principle, small enough perturbations in conductivity can be reconstructed accurately enough by considering just the linear problem. In EIT, starting from a known and usually homogeneous distribution *x*_*p*_, a set of measurement *V*_*p*_ is gathered. In sequence, a perturbation *δx* occurs causing a new *x* ≠ *x*_*p*_ and consequently a *V* ≠ *V*_*p*_. Calculating the Jacobian matrix (*J*) in which a computing method is introduced in [18] based on *x*_*p*_, the discrete form of the linear forward problem used in difference imaging becomes

(1) $$\begin{array}{c} J\delta x=\delta V\Leftrightarrow J\left(x_{p}-x\right)=V_{p}-V. \end{array}$$

In (1) only *V* is physically collected from the boundary of the volume as *V*_*p*_ is obtained by forward calculations:

(2) $$\begin{array}{c} J\left(x_{p}-x\right)=F\left(x_{p}\right)-V, \end{array}$$

in which *F*(*x*_*p*_) denotes the vector of simulated measurements derived from forward computations based on a model *x*_*p*_.

The Least squares method (LS) could be used to solve (2):

(3) $$\begin{array}{c} \underset{x_{p}-x}{\min}{||J\left(x_{p}-x\right)-\left[F\left(x_{p}\right)-V\right]||}_{2}. \end{array}$$

For the linear least squares problem, the Jacobian matrix *J* is very ill-conditioned and singular. This problem is remedied by regularizing the matrix *J* and solving a new problem that is well conditioned. A general version of Tikhonov regularization method is used:

(4) $$\begin{array}{c} \min\left\{{||J\left(x_{p}-x\right)-\left[F\left(x_{p}\right)-V\right]||}_{2}^{2}+\lambda {||L\left(x_{p}-x\right)||}_{2}^{2}\right\}, \end{array}$$

where *λ* is Tikhonov regularization parameter, and *L* is a matrix that defines a norm on the solution through which the “size” is measured. Often, *L* represents the first or second derivative operator. If *L* is the identity matrix, then the Tikhonov problem is said to be in standard form. So we can get the solution from (2)~(4):

(5) $$\begin{array}{c} \delta x=x_{p}-x={\left(J^{T}J+\lambda I\right)}^{-1}J^{T}\times \left[F\left(x_{p}\right)-V\right]. \end{array}$$

It has the effect of damping any large oscillations. A scaled identity matrix adding to the Jacobian matrix *J* makes the solution stable. But the condition number which indicates the sensitivity of the uncertainty is still large and the solution has the side effect of the smoothing caused by that identity matrix [9].

To reduce the condition number and side effect, we combined Tikhonov regularization with NOSER type regularization. In NOSER regularization, the regularization matrix is a simple diagonal weighting for *J* ^*T*^ *J* corresponding to the first and second difference operators. The equation is

(6) $$\begin{array}{c} \delta x={\left(J^{T}J+\varepsilon \times \mathrm{diag}\left(J^{T}J\right)\right)}^{-1}J^{T}\times \left[F\left(x_{p}\right)-V\right], \end{array}$$

where 0 < *ε* < 1 is the NOSER regularization parameter and diag⁡(*J* ^*T*^ *J*) denotes the diagonal matrix and also represents an approximation for the missing part of the second derivative of the mapping [19].

NOSER regularization works well in 2D field, but cannot correct the error caused by the noise in 3D model which is a very ill-posed problem. Tikhonov regularization could correct the error caused by weak noises and also has the side effect of the smoothing caused by that identity. If these two methods are combined, the condition number would be reduced (in Table 1), consequently, a better image will be obtained. The equation with combined regularization method can be written as

(7) $$\begin{array}{c} \delta x={\left(J^{T}J+\lambda I+\varepsilon \times \mathrm{diag}\left(J^{T}J\right)\right)}^{-1}J^{T}\times \left[F\left(x_{p}\right)-V\right]. \end{array}$$

As comparison and experiments between reconstructed results of different algorithms, including parameters choosing and discussions, have been made in previous work [20], the combined regularization was proved to be effective in eliminating errors and demonstrate better spatial resolution such as target's location and size.

### 2.3. Finite Element Mesh and Impedance Calculation

**Figure 2:**
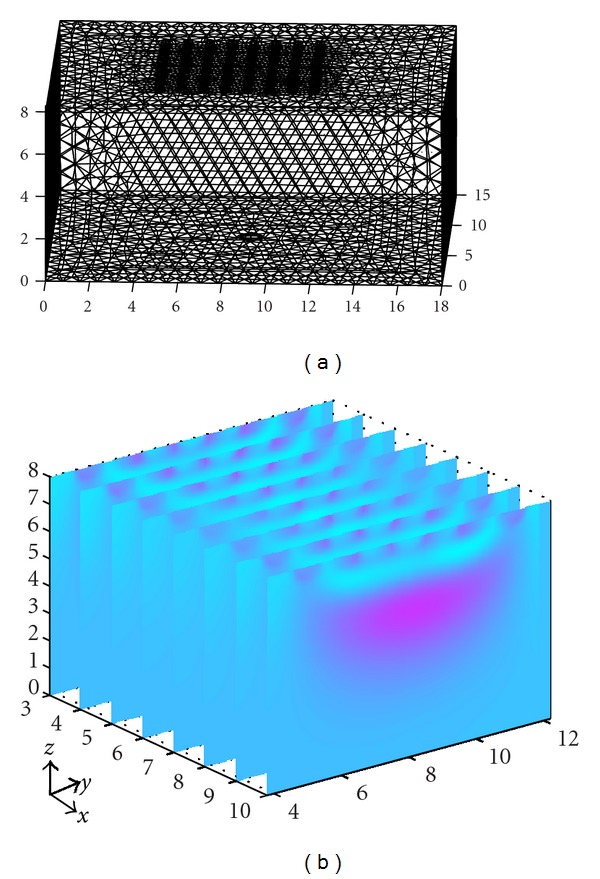
(a) Tetrahedral finite elements, (b) Electrical impedance slices by solving the inverse problem.

To calculate the discrete impedance within three-dimensional space, we first have to conduct tetrahedral finite element meshing in the whole measurement space as in Figure 2(a). As in the followed experiment, for example, the cuboid phantom was meshed into 79 307 finite elements with 14 876 nodes in space. Then, the Jacobian matrix could be obtained via complete electrode model by calculating the voltage of each node with analytical method [21]. The combined regularization matrix could be then deduced from Jacobian by choosing the proper parameters *λ* and *ε*. Finally, spatial distribution of the electrical impedance in the model could be approximated from the boundary conditions which were the voltage measurements from the electrodes. Although the accuracy of discrete impedance calculated from the combined regularization had been improved and large, there was still disturbance around the electrodes, as we could see from Figure 2(b). Another problem was that the electrical impedance changes gradually, in case that we were not able to draw the actual boundary of the anomaly buried in the background. Therefore, we had to develop a feasible and reliable way to eliminate the noise, sketch out the boundary of objects, and reconstruct images with boundary surface detection in 3D field.

### 2.4. Boundary Detection

In many cases, the gray-level-based decent isosurface of boundary surfaces can well separate voxels belonging to an object from voxels belonging to the background and therefore can be applied in the segmentation of 3D images [22]. However, the impedance approximations obtained from inverse problems are usually of high level noises which are not applicable for direct isosurface calculation. As the boundaries of the target object are usually across intensity values which have great differences from the background, they are actually steplike edge surfaces, defined as surface where great change of intensity value occurs. A volumetric image can be considered as the discrete sampling of the underlying three-dimensional continuous function at the grid points of the three-dimensional regular grid.

The boundary surface within the volumetric image can be considered as the implicitly defined continuous surface contained in the continuous sampling region of the volumetric image. Recall that, in a volumetric image, different structures usually correspond to different image intensities. Thus, the impedance intensities on either side of the boundary surface of the structure within a volumetric image have sharp changes. Such a boundary surface belongs to a steplike edge surface and therefore it is a continuous zero-crossing surface with a high gradient value. Mathematically, the boundaries within 3D image could be presented as follows [23]:

(8) $$\begin{array}{c} \nabla^{2}f\left(x,y,z\right)=0,\\ ||\nabla f\left(x,y,z\right)||\geq T, \end{array}$$

where *T* is a predetermined gradient threshold, ∇^2^ *f*(*x*, *y*, *z*) represents the Laplacian function of *f*(*x*, *y*, *z*), and ||∇*f*(*x*, *y*, *z*)|| represents the gradientmagnitude function of *f*(*x*, *y*, *z*). *T* can be selected by other methods that are used to select the gradient threshold in the edge detection of a 2D image [24].

Since the boundary surface of the volumetric image is determined, the subsequent processing can be performed to reduce the noises and improve the reconstruction quality, which will be further elaborated in the following sections.

### 2.5. Edge Elements Detection

The electrical impedance is sampled from tetrahedron grid as described above, and all such tetrahedrons form a continuous space occupied by 3D image. Steplike edge surfaces in the volume pass through, or are included in, some tetrahedrons. All tetrahedron elements are divided into two categories: those that are passed through by a steplike edge and the rest which are not. We first detect the edge elements and then compute the steplike edges in each edge element. For each edge element, since steplike edge passes through it, at least three of its edges are intersected by steplike edges. Without loss of generality, we assume that the edge linking vertexes of one tetrahedron are *P*_1_(*i*, *j*, *k*) and *P*_2_(*i* − 1, *j*, *k*). In terms of the literature [23], if the one side of the tetrahedron intersects with the edge surfaces, the two endpoints in the side of the section, respectively, having a high value of the gradient, and their Laplacian values, are of different signs.

In the edge elements, each edge intersected has the following characterizations:

1. both vertices have high gradient values: ||∇*f*(*P*_1_)|| + ||∇*f*(*P*_2_)|| ≥ 2*T*,
2. two vertices are a pair of zero-crossing points: ||∇^2^ *f*(*P*_1_)|| · ||∇^2^ *f*(*P*_2_)|| < 0.

Accordingly, by tagging the edge surfaces intersection as well as determining whether three edges of the tetrahedron are intersected by the edge surfaces, we will find out the edge elements and locate the edge surface from the three-dimensional images. 

### 2.6. Extraction and Reconstruction

Among the detected edge elements, there are true elements which contain the edge surfaces, besides pseudoedge elements which caused by the noise and object details. The pseudo-edge elements, usually just a small collection of interlinked tetrahedron, and the tetrahedrons which include the edge surfaces, are typically a relatively large collection according to the characteristics in bladder filling process. Therefore, judging by whether the edge elements are coplanar, we can extract the larger connected set by removing the small ones. 

**Figure 3:**
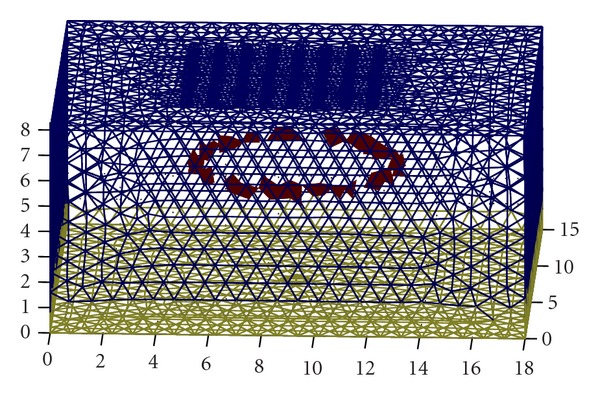
Extracted edge elements from tetrahedron slice.

All the tetrahedron elements in the model are divided into slices in horizontal, each slice contains incomplete seed elements representing the edge surfaces (in Figure 3), from which the edge surface of the object can be tracked. If the edge surface intersects with one face of an edge element, the adjacent tetrahedron of mutual face of that one is inevitable an edge element as well. This definition originated in the region growing method, the algorithm using 3D region growing method [25]. By checking the adjacent elements which meet the coplanar criteria, the seed grows in the original area, until the target area does not grow any longer. In virtue of this property, we can track and recombinant the most similar edge elements to the objects, by which are not detected yet, starting from the determined seed elements. 

Each edge element contains a piece of edge surface which is in fact the isosurface of zero value of the Laplacian function in three-dimensional image. By computing the zero-crossing surface of the Laplacian in each edge element, eventually, a triangulated model of steplike edges is obtained. From each edge element, the surface patch could be extracted by using the Marching Cubes and its improved algorithm as the polygonal approximation. This algorithm guarantees that the surface patches extracted from the adjacent edge elements can be spliced together and constitute a polygonal surface model [26].

## 3. Results and Discussion

### 3.1. Experiment Platform

**Figure 4:**
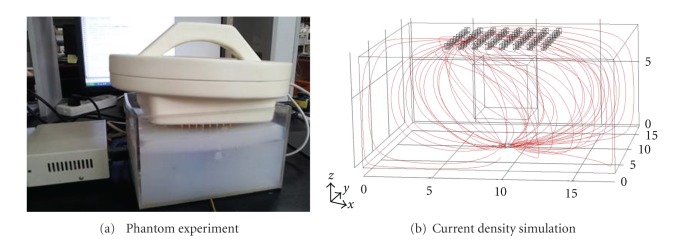


The process of imaging reconstruction was illustrated and verified by applying the algorithm on a dataset obtained from an experimental feasibility trial. The cuboid phantom was made of polycarbonate, with 18 cm long, 15 cm wide and 10 cm high as in Figure 4(a). The 8 × 8 electrodes array, of each electrode diameter 4 mm and gap of 8 mm between each, was placed on the upper surface and the back electrode as the ground was placed on the center of lower surface. The current density simulation model was represented in Figure 4(b), and from which we could configure that the current density in the middle area below the electrode array was larger which indicated higher sensitivity. 

The experiment was carried out by using agar of 0.1 S/m (at 200 kHz) as the background with a different size of cuboid hole in the middle of background as the inclusion (detailed in Section 3.2). The hole was filled with saline solution dropped of India ink, which conductivity measures as 0.892 S/m by the Mettler-Toledo hand-held portable liquid conductivity measurement instrument SG7, then it was covered with 1.5 cm thick of 0.1 S/m agar same as the background. So the saline solution was wrapped in agar to simulate the urine in the bladder.

As the conductivity of saline varies with the frequency, a relatively sharp change in order to distinguish it from the agar happens at the frequency around 200 kHz [27]. Furthermore, there is also a strict regulation to limit the injection current into the body, which is less than 10 mA of frequency at 100 kHz or higher frequency [28]. Accordingly, we set the current waveform frequency 200 kHz with the amplitude of 10 mA. The injection-and-measurement strategy was that of Section 2.1 described.

### 3.2. Preliminary Data Analysis

**Figure 5:**
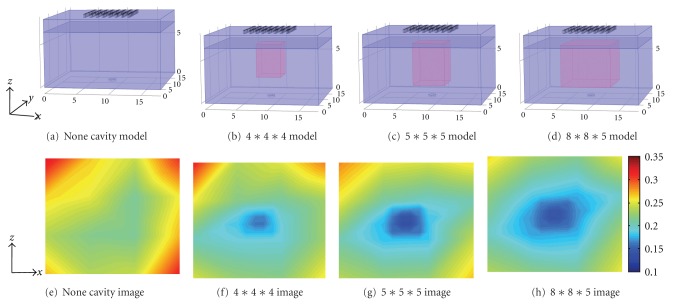
Experiment models and the corresponding 2D images.

A completely full bladder of human is capable of holding approximately 1 liter of fluid. However, the urge to urinate ordinarily occurs when the bladder contains about 200 mL of urine, which value should be smaller considering of the age and body figure [29]. So we chose different volume of saline solution to simulate the urine in the experiment, as in Figures 5(a)–5(d) in red. They were, respectively, 0 mL in Figure 5(a), 4 cm∗4 cm∗4 cm that was 64 mL in Figure 5(b), 5 cm∗5 cm∗5 cm that was 125 mL in Figure 5(c), and 8 cm∗8 cm∗5 cm that was 320 mL in Figure 5(d). As long as these volumes are able to be estimated, we are able to predict the right moment to micturate or whether the volume reaches the critical value. 

Figures 5(e)–5(h) illustrate the 2D image projection from direct acquisition data without any algorithm applied. That means the color of image reflects the voltage which corresponds to the impedance from the measurement electrodes. We can figure out that the results are with perturbations at the corners due to the edge effect of container in Figures 5(e) and 5(f). As the volume increases in Figures 5(g) and 5(h), the blue area indicating the lower impedance increases, but obviously, the shapes are irregular without any depth information. As a result, it is ambiguous for diagnose which inspirit us to improve the results by utilizing more optimized methods and algorithms.

### 3.3. Three-Dimensional Reconstruction and Discussion

**Figure 6:**
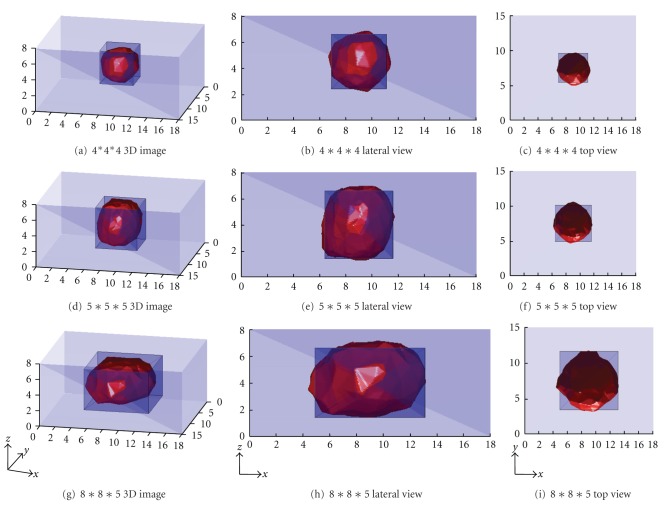
3D reconstructions from different volumes of saline solution.

The 3D representations of the reconstructed perturbation from the experiments were shown above.  Figure 6(a) was the reconstructed image from 4 cm∗4 cm∗4 cm saline solution, Figures 6(b) and 6(c) are the corresponding lateral and top views. From those of which we could figure out that the reconstructed target's location was basically identical, whereas the shape of target transferred from cubic to round-like. It was by reason of the regularization-based algorithms applied the least squares method, which is different from back-projection [7] and genetic algorithms [10], and approached the perturbation generally, in case that the sharp changes were smoothened and some object details were as well omitted. Reconstructions from other two volumes in Figures 6(d)–6(i) reflected the similar characteristic. In addition, we could point out that while the volume increased, the distortion was getting more serious, because of the sensor array was getting comparatively smaller in contrast of the volume. As a result, the sensitivity of the algorithm decreased and the image deteriorated because of incomprehensive boundary conditions.

**Figure 7:**
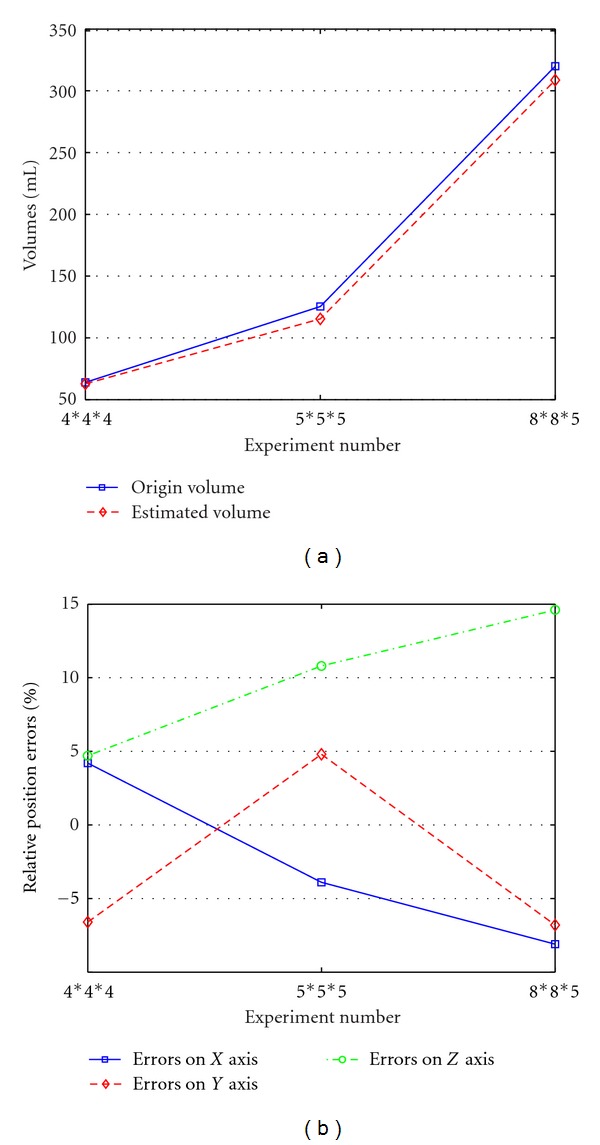
(a) Volume comparison of models and (b) Relative position errors.

To estimate the object volume precisely, the tank model was gridded by the interval of 1 mm in *x*, *y*, *z* coordinates. We could judge the entire space being divided into 180 × 150 × 80 = 2,160,000 cubes. Each cube volume of 1 × 10^−3^ mL, and each node from the cubes could be surrounded in the edge surface or not. Here, we defined the cube which has at least 4 noncoplanar vertices in the edge surface considered to be a valid one. As in our experiment, the number of included cubes in Figure 6(a) was 62,783, the number of Figure 6(d) was 115,429, and Figure 6(g) was 307,725. The corresponding estimated volumes were approximately 62.8 mL, 115.4 mL and 308.7 mL. Comparing with the origin volume with estimation, Figure 7(a) reveals that the estimation of model 4∗4∗4 is almost identical to that of actual volume, even though their shapes are inconsistent. Whereas the 5∗5∗5 and 8∗8∗5 models' estimation are lower than that of real ones, the volumetric errors are less than 10% which was still at acceptable level.

The position of an anomaly is defined to be located as the centre of mass of HA set in reconstruction images as

(9) $$\begin{array}{c} \text{position}=\frac{\sum_{m}\sigma_{m}\cdot p_{m}}{\sum_{m}\sigma_{m}}. \end{array}$$

Here, *p*_*m*_ is the position vector (*x*_*m*_, *y*_*m*_, *z*_*m*_) within the domain. Position error is a figure of merit defined as the proportional difference in position of the centre of mass of the reconstructed image HA set and the centre of mass of the generating anomaly. The smaller PE indicating the reconstructed image is more approximate to the center of the target object.

As we consider the conductivity of saline solution to be homogeneous, the centre of origin should be at the center of the cuboids. In terms of position error in Figure 7(b), the configurations show that relative position errors are various at different axes. Although the result indicates nonlinearity in different models, the error percentages are generally between −8%~8%. We could also figure out that, the errors on *z*-axis in green is comparatively greater than that of *x*- and *y*-axis which exceed of 10%. It is because of the higher sensitivity as the object closer to the electrode array, this is resulting from both the current density distribution and the finite elements at the top are smaller than the bottom in our algorithm. Moreover, we realize that, as the size of target increase especially for the model 8∗8∗5, the target is getting closer to the edge of the electrode array, namely, the position errors increase.

By utilizing the combined regularization algorithm and integrating with edge elements filtering and rearrangement, 3D images were able to display for qualitative image evaluation. The differences between the objectives and background from reconstructions were significant. By and large, the target's locations were easy to be distinguished, nevertheless the reconstructed object did not directly correspond with the exact shape of the original. This experiment demonstrated that it was possible to obtain and localize reliable 3D images of conductivity changes, employing 65 channels, the result presented to be superior to that of traditional methods and of considerably highly approximation to the target.

## 4. Conclusions

This paper addresses the issue of presenting a method for EIT 3D reconstruction and targets identification, by which aim to predict the urine volume in bladder. The performance of the proposed algorithms has been investigated and demonstrated by mathematical exposition. The approximation problem of boundary surface within 3D images is also described, it is applied not only to reduce the system noise which leads to holes, protrusions, and debris in reconstruction, but also deliver us readily to identify and calculate visual images. The reconstruction images provide more information as well, including depth and volume, and contrast from the background. 

Overall, EIT image reconstruction is a nonlinear and ill-posed inverse problem of spatially variant estimation. Uncertainties caused by these properties prevent EIT images from having high resolution. These preliminary results indicate that sufficient finite element modeling of the impedance distribution in the abdomen, proper inverse problem, and tracking algorithms choosing enable this technology to be applicable for routine measurement of bladder volume.

This approach is convenient to apply with image reconstructions that are spatially variant, which promises to deliver a joint distribution and material identification and estimates in a single measurement process. It yields an alternative method for reporting the bladder filling so that instead of reporting in terms of pressure and ultrasound images, we may be able to present clinicians medical visualization and extract certain boundary surface structure.

## Figures and Tables

**Table 1:** Typical condition number of Jacobian matrix and regularization algorithms.

	Jacobian matrix	Tikhonov regularization	NOSER regularization	Combined regularization
Condition number	1.536 × 10^16^	1.109 × 10^11^	3.854 × 10^9^	9.294 × 10^8^
